# Primary Hepatic Extranodal Marginal Zone Lymphoma of Mucosa-Associated Lymphoid Tissue in a Patient with Chronic Hepatitis B Virus Infection: Case Report and Summary of the Literature

**DOI:** 10.3390/medicina57030280

**Published:** 2021-03-18

**Authors:** Yuki Yamashita, Satoru Joshita, Hiroyuki Kobayashi, Shun-ichi Wakabayashi, Ayumi Sugiura, Tomoo Yamazaki, Takeji Umemura

**Affiliations:** 1 Division of Gastroenterology and Hepatology, Department of Medicine, Shinshu University School of Medicine, Matsumoto 390-8621, Japan; unagichazuke.sansho.3694@gmail.com (Y.Y.); h_kobayashi1980@yahoo.co.jp (H.K.); shun_1@me.com (S.-i.W.); a19860530@gmail.com (A.S.); ymzktm6@shinshu-u.ac.jp (T.Y.); tumemura@shinshu-u.ac.jp (T.U.); 2 Department of Life Innovation, Institute for Biomedical Sciences, Shinshu University, Matsumoto 390-8621, Japan

**Keywords:** primary hepatic MALT lymphoma, chronic hepatitis B virus infection, contrast-enhanced ultrasonography

## Abstract

Background: The incidence of extranodal marginal zone lymphoma of mucosa-associated lymphoid tissue (MALT lymphoma) is low, at 7–8% of all non-Hodgkin lymphoma cases. The most common site of MALT lymphoma occurrence is the stomach. Primary hepatic extranodal marginal zone lymphoma of MALT is classified as a type of non-gastric MALT lymphoma and is considered extremely rare, with no consensus on imaging study findings or treatment due to a limited number of reports. We herein describe a rare case of primary hepatic extranodal marginal zone lymphoma of MALT with underlying hepatitis B infection (HBV) and present useful diagnostic findings of various imaging modalities, including contrast-enhanced ultrasonography (CEUS) with Sonazoid. Case presentation: A 66-year-old woman was diagnosed as being a non-active carrier of HBV at 51 years of age at the time of total hysterectomy and bilateral adnexectomy for uterine cervical cancer. She was admitted to our hospital following the incidental detection of two focal liver lesions on computed tomography. The lesions were considered malignant based on clinical and other radiologic imaging findings. Her CEUS results of hypo-enhancement in the portal and late phases were consistent with those of previously reported cases of hepatic extranodal marginal zone lymphoma of MALT, and histological liver biopsy findings were compatible with the diagnosis. Conclusions: Primary hepatic extranodal marginal zone lymphoma of MALT is a rare condition that can appear in HBV carriers. Characteristic CEUS findings may help in disease diagnosis. Clinicians should bear primary hepatic extranodal marginal zone lymphoma of MALT in mind when encountering patients with focal liver lesions which exhibit image findings different from those of typical hepatocellular carcinoma.

## 1. Introduction

Extranodal marginal zone lymphoma of mucosa-associated lymphoid tissue (MALT lymphoma) can appear in a number of epithelial tissues. The incidence of MALT lymphoma is low at 7–8% of all non-Hodgkin lymphoma cases [1]. The stomach is the most common site of MALT lymphoma development in patients with chronic gastritis induced by *Helicobacter pylori* (HP) infection [2]. Other reported causes of MALT lymphoma include chronic autoimmune diseases and hepatitis C virus (HCV) infection [3,4,5], suggesting an involvement of chronic inflammation in MALT lymphoma onset.

In addition to gastric MALT lymphoma, non-gastric MALT lymphomas affecting the skin, conjunctiva, orbit, salivary glands, thyroid, upper airways, lung, breast, and urinary tract have been reported [6]. Primary hepatic extranodal marginal zone lymphoma of MALT is a rare subtype of hepatic malignant lymphoma [7], the incidence of which is low [6,8]. Although the etiology of primary hepatic MALT lymphoma remains unknown, cases with such accompanying chronic liver conditions as hepatitis B virus (HBV) [7,9,10,11,12,13,14,15], HCV [16,17,18,19,20,21,22,23], alcohol liver disease [24], non-alcoholic steatohepatitis [25], primary biliary cholangitis [26,27], and autoimmune hepatitis [28,29] have implicated chronic inflammation in disease development. Histological liver assessment is required for the accurate diagnosis of primary hepatic extranodal marginal zone lymphoma of MALT. However, no consensus on image study findings has been established due to a limited number of reported cases.

We herein describe a rare case of primary hepatic extranodal marginal zone lymphoma of MALT with underlying HBV and present useful diagnostic findings on imaging modalities, including contrast-enhanced ultrasonography (CEUS) with Sonazoid, along with a review of the literature.

## 2. Case Presentation

A 66-year-old woman was diagnosed as being a non-active carrier of HBV at 51 years of age at the time of total hysterectomy and bilateral adnexectomy for uterine cervical cancer. Abdominal non-contrast computed tomography (CT) was performed yearly as follow-up. At the age of 66 years, she was referred to our department for further evaluation of hypo-dense lesions of 3.5 cm in diameter at S8 and 3 cm in diameter at S5 in the liver. She had no subjective symptoms, including fever, night sweats, or weight loss. She was under medical treatment with olmesartan and medoxomil for hypertension and eldecalcitol for osteoporosis. She had no allergies but had smoked 5 cigarettes per day for 46 years and habitually drank 20 g of ethanol daily. She had no particular family history of HBV infection or other chronic liver disease. No abnormal findings were noted on her physical examination. Laboratory tests revealed no hepatic enzyme elevation, although serologic testing for HBV was positive (HBs antigen positive, HBe antigen negative, HBe antibody positive, HBV DNA 2.6 Log IU/mL). α-fetoprotein and protein induced by vitamin K absence or antagonist II were within the upper limits of normal, while soluble interleukin-2 receptor was slightly elevated at 453 U/mL (Table 1).

**Figure 1:**
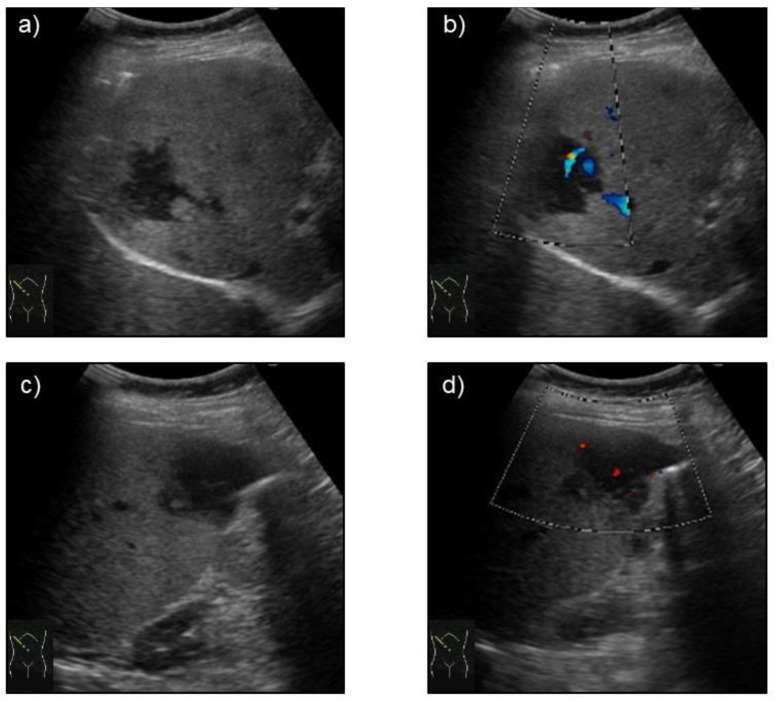
B-mode ultrasonography depicted a hypo-echoic tumor lesion at segment S8 in the liver (**a**), through which a penetrating vessel was visualized by color Doppler imaging (**b**). B-mode ultrasonography showed a hypo-echoic tumor lesion at segment S5 in the liver (**c**), through which a penetrating vessel was visualized by color Doppler imaging (**d**).

**Figure 2:**
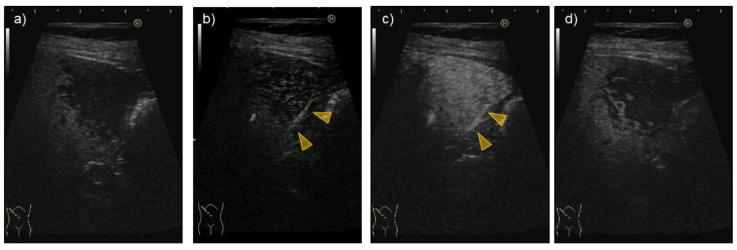
The tumor lesion at segment S5 in the liver was hypo-echoic on B-mode ultrasonography (**a**), through which a penetrating vessel was detected (**b**, arrowheads), which was enhanced in the arterial phase (**c**, arrowheads). The vessel was evident in the Kupffer phase (**d**) on enhanced ultrasonography using Sonazoid.

**Figure 3:**
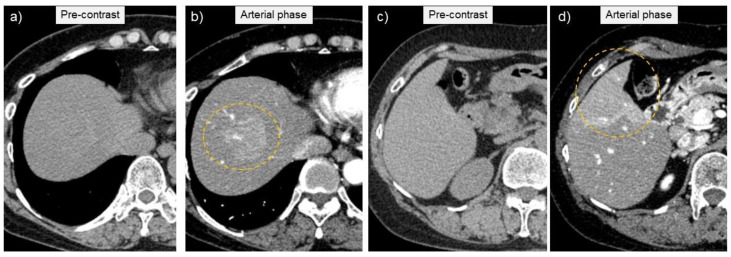
Computed tomography depicted tumor lesions at segments S8 (**a**) and S5 (**c**) in the liver as low and iso-density lesions in pre-contrast imaging, both of which are seen as ring-enhancement pattern lesions in the arterial dominant phase on contrast-enhanced imaging (circles; (**b**): S8 lesion, (**d**): S5 lesion).

**Figure 4:**
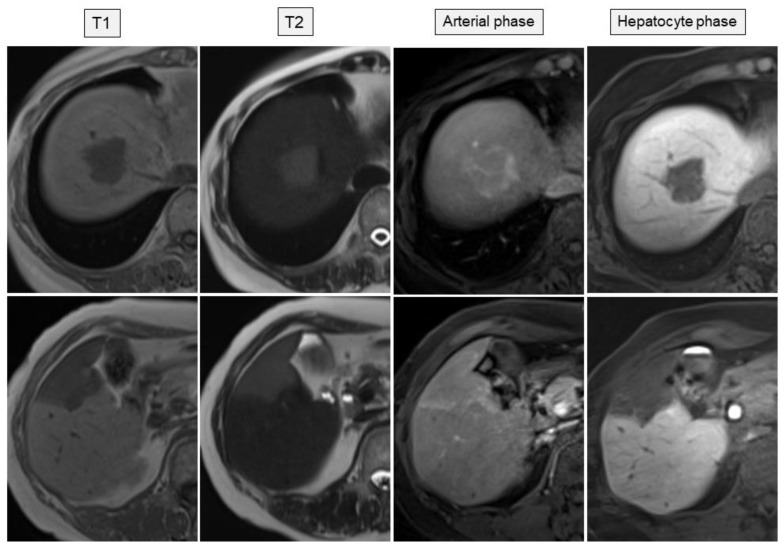
Gd-EOB-DTPA-enhanced magnetic resonance imaging visualized the lesions at segments S8 (upper panel) and S5 (lower panel) in the liver as low-intensity lesions in T1-weighted images, high-intensity lesions in T2-weighted images, ring-enhancement pattern lesions in the arterial phase, and mainly low intensity lesions in the hepatobiliary phase.

Abdominal ultrasonography (US) using HI VISION Preirus (Hitachi, Ltd., Tokyo, Japan) displayed hypoechoic lesions of 3.5 cm in diameter at S8 and 3 cm in diameter at S5 in the liver (Figure 1), through which penetrating vessels were depicted by color Doppler US (Figure 1). The S5 tumor was depicted as a homogeneously hyper-enhanced lesion with penetrating vessels in the early arterial phase after intravenous Sonazoid injection, which began to wash out in 15 seconds. The tumor was hypo-enhanced in the portal and late phases on CEUS (Figure 2). Both the S8 and S5 lesions were seen as low and iso-density areas in pre-contrast images and as having a ring-enhancement pattern in the arterial dominant phase on dynamic CT (Figure 3). The lesions were also depicted as having low intensity on T1-weighted magnetic resonance imaging (MRI) and hyper-intensity on T2-weighted MRI. Gd-EOB-DTPA-enhanced MRI showed ring enhancement pattern lesions in the arterial phase and mainly low intensity lesions in the hepatobiliary phase (Figure 4).

**Figure 5:**
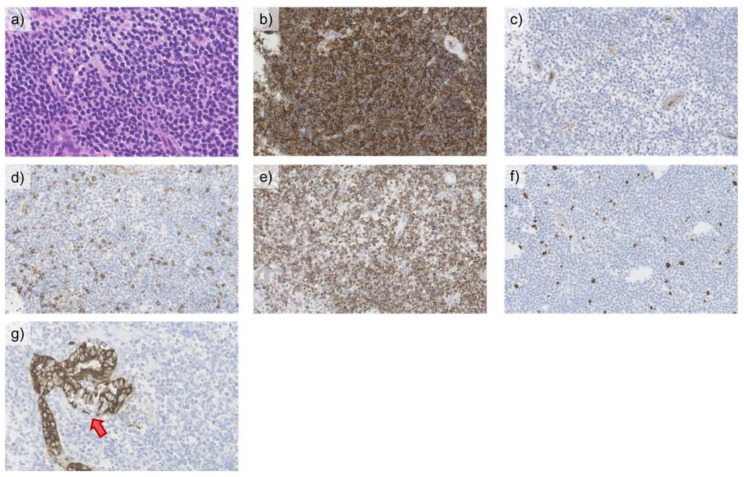
A punched biopsy of the segment S8 tumor lesion revealed the infiltration of small atypical lymphoid cells (**a**) (hematoxylin and eosin staining, ×400 magnification), which were CD20+ (**b**), CD10- (**c**), CD5- (**d**), and Bcl2+ (**e**) by immunohistochemistry (×200 magnification). The Ki-67 labeling index was calculated as approximately 5% (×200 magnification) (**f**). Cytokeratin (AE1/AE3) was stained on the lymphoepithelial lesions (**g**, arrow) (×200 magnification).

A tumor tissue specimen taken by percutaneous US-guided liver biopsy revealed the histological findings of diffuse infiltration of small atypical lymphoid cells with slightly irregular nuclear contour, dense chromatin, and moderately abundant pale cytoplasm, all of which were consistent with malignant lymphoma. The tumor cells were CD20+, CD10-, CD5-, and Bcl2+, with a Ki-67 labeling index of 5%. Cytokeratin (AE1/AE3) staining was also evident on the lymphoepithelial lesions (Figure 5). The amount of parenchymal non-tumoral liver tissue was insufficient to histologically assess the degree of inflammation and fibrosis.

**Figure 6:**
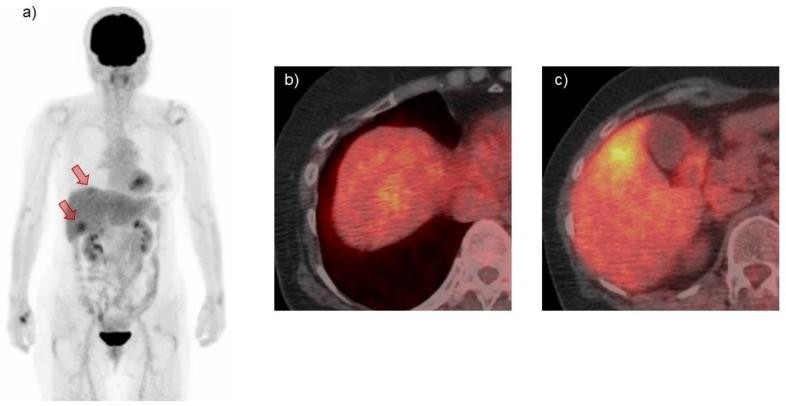
FDG-PET and computed tomography detected increased uptake in the liver tumor lesions (arrows), with no uptake in other organs (**a**–**c**).

No primary or secondary MALT lymphoma lesions were detected by upper and lower gastrointestinal endoscopy. A bone marrow biopsy revealed no signs of MALT lymphoma invasion, although the t (11;18) (q21; q21) API2/MALT1 translocation was noted. 18F-fluorodeoxyglucose-positron emission tomography (FDG-PET) showed increased uptake in the liver tumors, with none in other organs (Figure 6). Accordingly, she was diagnosed as having primary hepatic extranodal marginal zone lymphoma of MALT of clinical stage II as defined by the Ann Arbor criteria [30]. She declined chemotherapy with rituximab, opting instead for oral tenofovir alafenamide (TAF) for her chronic HBV infection and close follow-up.

## 3. Discussion

Primary hepatic extranodal marginal zone lymphoma of MALT is a very rare neoplasm [6,31], histologically confirmed in only 3% of non-gastric MALT lymphoma patients [6]. Due to its scarcity, little is known on the epidemiology, clinical features, radiological investigation, and optimal treatment of this condition, as summarized in Table 2 [7,29].

Since there are few reported cases of hepatic extranodal marginal zone lymphoma of MALT that include CEUS findings [32,33], the entity’s typical CEUS characteristics have not yet been defined. In the present case, both S8 and S5 tumors were homogeneously hyper-enhanced in the arterial phase and hypo-enhanced in the portal and late phases on CEUS, which were consistent with previous reports. The tumors also showed penetrating vessels in the arterial phase. It was discovered earlier that multiple vascular channels could often be seen coursing through hepatic malignant lymphoma, including MALT lymphoma, which were referred to as the “vessel penetration sign” [34,35]. Comparisons of the image results of the present case with previously reported extranodal marginal zone lymphoma of MALT findings and typical hepatocellular carcinoma are summarized in Table 3. Based on the current literature, hepatic extranodal marginal zone lymphoma of MALT should be included in the differential diagnosis if the vessel penetration sign is observed. However, it is noteworthy this sign is sometimes present in liver metastatic tumors and cholangiolocellular carcinoma as well [36,37]. Histological assessment is therefore needed to distinguish among malignant tumors.

We searched the English literature published between 1995 and 2020 using the terms “mucosa-associated lymphoid lymphoma” and “hepatitis B virus” in PubMed/MEDLINE. Fourteen cases were extracted and summarized in Table 4. There were five cases in HBV carriers, four in chronic hepatitis, and one in liver cirrhosis. Although hepatic extranodal marginal zone lymphoma of MALT usually develops in the presence of chronic inflammation [7,38], we considered that the hepatic extranodal marginal zone lymphoma of MALT in the present case was associated with an HBV carrier state. Significant inflammation and fibrosis have been reported in 37% of HBV carriers [39]. However, our patient’s non-tumoral liver tissue specimen was insufficient to assess the degree of inflammation and fibrosis. Further studies are needed on the impact of an HBV carrier state on the development of hepatic extranodal marginal zone lymphoma of MALT.

Lastly, no optimal treatment strategies for primary hepatic extranodal marginal zone lymphoma of MALT have been established to date. Most reported cases were treated by surgical resection and/or chemotherapy containing rituximab. Two cases were closely observed under conservative treatment. As the eradication of HP results in long-term remission in up to 80% of patients for gastric MALT lymphoma, it should be considered the first treatment step regardless of disease stage [2,40]. The clinical efficacy of direct-acting antivirals has also been documented in patients with HCV-associated non-Hodgkin lymphoma for viral eradication therapy [20,22]. Accordingly, we treated our patient with a nucleotide analog of TAF, which is a key drug in anti-HBV treatment, to suppress HBV-related chronic inflammation in the liver. Careful follow-up is needed to monitor the changes in HBV and hepatic extranodal marginal zone lymphoma of MALT.

**Table 3:** Comparison of image findings among the present case, previously reported cases, and typical hepatocellular carcinoma.

	Present Case	Common Findings of Previously Reported Cases [7,32,33,41,42]	Common Findings of Typical Hepatocellular Carcinoma [43,44,45]
US	Hypoechoic	Hypoechoic	Presence of halo and mosaic signs
CEUS	Arterial phase: hyper-enhanced Portal/late phase: hypo-enhanced	Arterial phase: hyper-enhanced Portal/late phase: hypo-enhanced	Arterial phase: hyper-enhanced Portal/late phase: hypo-enhanced
CT	Low and iso-density	Low and iso-density	Low/iso-/high density
Dynamic CT	Ring enhancement pattern	Enhanced/ring enhancement pattern/not enhanced	Enhanced and washout
MRI	T1-weighted: low intensity, T2-weighted: high intensity	T1-weighted: low intensity, T2-weighted: high intensity	T2-weighted: high intensity
Gd-EOB-MRI	Enhanced in arterial phase, low intensity in hepatocyte phase	Enhanced in arterial phase, low intensity in hepatocyte phase	Enhanced in arterial phase, low intensity in hepatocyte phase
PET	Abnormal accumulation	Abnormal accumulation	Nearly half of cases have no accumulation

Abbreviations: CEUS, contrast-enhanced ultrasonography; CT, computed tomography; MRI, magnetic resonance imaging; PET, positron emission tomography.

**Table 4:** Reported cases of primary hepatic extranodal marginal zone lymphoma of MALT lymphoma with HBV.

Age (Years)	Sex	HBV	Treatment	Follow-Up (Months)	Reference
37	M	CH	R-CHOP	N/A	[15]
73	M	Carrier	Resection	6	[7]
59	F	CH	Observation	N/A	[14]
52	F	N/A	R-CVP	60	[42]
54	M	N/A	R-CVP, RT	36	[41]
65	M	N/A	Chemotherapy	24	[41]
80	M	N/A	Chemotherapy	15	[41]
57	F	N/A	Resection, rituximab	72	[46]
50	F	CH	Observation	32	[47]
59	M	CH	Resection	48	[13]
53	M	Carrier	Resection, R-CHOP	N/A	[12]
38	M	Carrier	Resection, CHOP	15	[11]
59	M	LC	Liver transplantation	6	[10]
36	M	Carrier	Resection, rituximab	40	[9]
66	F	Carrier	NUC, observation	Ongoing	Present case

Abbreviations: CH, chronic hepatitis; LC, liver cirrhosis; N/A, not applicable; NUC, nucleotide analog; R-CHOP, rituximab + cyclophosphamide + doxorubicine + vincristine + prednisone; R-CVP, rituximab + cyclophosphamide + vincristine + prednisone; RT, radiotherapy.

## 4. Conclusions

Primary hepatic extranodal marginal zone lymphoma of MALT is rare among non-Hodgkin lymphomas. Clinicians should bear this disease in mind when encountering patients with focal liver lesions presenting image findings different from those of typical hepatocellular carcinoma along with the vessel penetration sign. CEUS may be of assistance in the identification of primary hepatic extranodal marginal zone lymphoma of MALT. Further studies are needed to investigate the clinical utility of chronic inflammation suppression in patients with primary hepatic extranodal marginal zone lymphoma of MALT associated with HBV infection.

## Figures and Tables

**Table 1:** Laboratory data on admission.

Hematology			Chemistry			Tumor Markers		
While blood cells	5503	/μL	Total protein	7.5	g/dL	AFP	4.5	ng/mL
Neutrophils	63.5	%	Albumin	4.8	g/dL	PIVKA-II	17	mAU/mL
Lymphocytes	28	%	AST	20	U/L	CEA	1.6	ng/mL
Red blood cells	447 × 10^4^	/µL	ALT	17	U/L	CA19-9	39.3	U/mL
Hemoglobin	13.9	g/dL	LDH	232	U/L	CA12-5	18	U/mL
Platelet count	24.1 × 10^4^	/μL	ALP	172	U/L	CA15-3	12.8	U/mL
<Coagulation>			GGT	32	U/L	sIL-2R	385	U/mL
Prothrombin%	127.3	%	T-Bil	0.82	mg/dL	<Infection markers>		
APTT	25.4	s	BUN	15	mg/dL	HBs-Ag	70.6	IU/mL
			Cre	0.76	mg/dL	HBe-Ag	0.1	C.O.I
			Na	141	mEq/L	HBe-Ab	100	%INH
			K	4.4	mEq/L	HBV DNA	2.6	Log IU/mL
			CRP	0.04	mg/dL	HCV-Ab	(-)	

Abbreviations: AFP, alpha-fetoprotein; ALT, alanine aminotransferase; ALP, alkaline phosphatase; APTT, activated partial thromboplastin time; AST, aspartate aminotransferase; BUN, blood urea nitrogen; CA19-9, carbohydrate antigen 19-9; CA12-5, carbohydrate antigen 12-5; CA15-3, carbohydrate antigen 15-3; CEA, carcinoembryonic antigen; Cre, creatinine; CRP, C-reactive protein; GGTP, gamma-glutamyltranspeptidase; LDH, lactate dehydrogenase; T-Bil, total bilirubin; PIVKA-II, protein induced by vitamin K absence or antagonist-II; sIL-2R, soluble interleukin-2 receptor.

**Table 2:** Epidemiology, clinical features, radiological investigation, and treatment of hepatic extranodal marginal zone lymphoma of MALT.

Epidemiology	Predominantly in elderly individuals in their 60’s at a similar sex ratio
Clinical features	No symptoms, abdominal discomfort, poor appetite, gastrointestinal symptoms, B symptoms (fever, weight loss, and night sweats)
Radiological investigation	US, CEUS, CT, MRI, PET-CT
Treatment	Surgery, chemotherapy, radiotherapy, combined therapy

## Data Availability

The datasets used and/or analyzed during the current study are available from the corresponding author on reasonable request.
